# Improving miRNA-mRNA interaction predictions

**DOI:** 10.1186/1471-2164-15-S10-S2

**Published:** 2014-12-12

**Authors:** Daniel Tabas-Madrid, Ander Muniategui, Ignacio Sánchez-Caballero, Dannys Jorge Martínez-Herrera, Carlos Oscar S Sorzano, Angel Rubio, Alberto Pascual-Montano

**Affiliations:** 1 National Center for Biotechnology-CSIC. Darwin 3. 28049, Madrid, Spain; 2 CEIT and TECNUN, University of Navarra, San Sebastián, Spain

**Keywords:** miRNA target prediction, bioinformatics, gene expression

## Abstract

**Background:**

MicroRNAs are short RNA molecules that post-transcriptionally regulate gene expression. Today, microRNA target prediction remains challenging since very few have been experimentally validated and sequence-based predictions have large numbers of false positives. Furthermore, due to the different measuring rules used in each database of predicted interactions, the selection of the most reliable ones requires extensive knowledge about each algorithm.

**Results:**

Here we propose two methods to measure the confidence of predicted interactions based on experimentally validated information. The output of the methods is a combined database where new scores and statistical confidences are re-assigned to each predicted interaction. The new scores allow the robust combination of several databases without the effect of low-performing algorithms dragging down good-performing ones. The combined databases obtained using both algorithms described in this paper outperform each of the existing predictive algorithms that were considered for the combination.

**Conclusions:**

Our approaches are a useful way to integrate predicted interactions from different databases. They reduce the selection of interactions to a unique database based on an intuitive score and allow comparing databases between them.

## Background

MicroRNAs (miRNAs) are a novel class of endogenous, ~22 nt long RNAs that post-transcriptionally regulate gene expression [1]. They guide the RNA-induced silencing complex (RISC) to their mRNA targets by sequence complementarity. In animals, miRNAs generally bind to the 3'UTR of the mRNA imperfectly and in most cases lead to translational inhibition of its targets [2]. In plants, most miRNAs match perfectly to the coding region of their targets causing mRNA degradation [3]. However, some other interactions have been identified in vitro, e.g. interactions with the 5'UTR and with the coding region of transcripts in mammals, with the 3'UTR of plants transcripts and even some expression-enhancing miRNAs[4].

MiRNAs are known to be involved in development [1], cell proliferation [5] and differentiation [6], apoptosis [7], cell cycle progression [8], tumorigenesis[9], and many other physiological and pathological processes[10].

There are several experimentally defined rules of miRNA targeting in mammals. Sequence complementarity with the "seed", generally the nucleotides 2 to 7, is sufficient to produce the repression of most animal mRNAs [5,11]. The seed matches are grouped into four canonical types: 6mer, 7mer-m8, 7mer-A1 and 8mer. There are also other features outside this region. In fact, sequence complementarity to nucleotides 13-16 of the miRNA can either enforce the affinity (supplementary pairing) or compensate for an incomplete seed pairing (complementary pairing). Furthermore, G:U wobbles within the seed are unfavorable to the regulation by miRNAs[12]. The thermodynamic stability of the duplex is a crucial feature of the interaction [13]. There are few other specificities for the mode of action of miRNAs in plants and metazoa[14].

Development of deep-sequencing methods have increased considerably the number of newly discovered miRNAs[15]. MirBase[16] is up to date the most complete database of precursor and mature miRNAs. Its latest update (release 20, June 2013) covers 206 species and contains 30,424 precursors and 24,521 microRNAs.

Presently there is a plenty of algorithms and databases to predict miRNA-mRNA interactions based on sequence, physical-chemistry properties, expression levels or even experimental validations. For a common molecular biologist this panorama represents yet another level of complexity to its every day work since there is no single answer to the questions of what are the target genes for a single miRNA? Which prediction algorithms or databases is the best performing one? How can I reduce the number of predicted targets? The nature of the questions reflects the current panorama. In this work we try to provide answers to those questions by proposing a methodology that combines and re-score the miRNA-mRNA interactions from all different available sources. Our intention is to provide the community with a unique source for miRNA-mRNA interactions based on the goodness of all available ones.

### miRNA-mRNA interactions

**Experimentally validated interactions**. Currently there are several databases with experimentally-validated interactions such as: miRWalk[17], miRecords[18], TarBase[19], miRTarBase[20] and starBase[21]. They differ mainly on the number of interactions they host. The starBase database includes interactions validated only by HITS-CLIP and by Degradome Sequencing. These techniques provide more accurate information about direct miRNA-mRNA interactions, and also about the exact binding site [22]. Table 1 contains detailed information about databases and number of interactions per organism.

**Table 1:** *Number of experimentally validated interactions*.

	Mirtarbase	Tarbase	Mirwalk	Mirecords
***Caernorhabditis elegans***	30	-	-	17
***Drosophila melanogaster***	115	-	-	81
***Danio rerio***	102	-	-	32
***Gallus gallus***	16	-	-	-
***Homo sapiens***	2860	878	5668	1276
***Mus musculus***	537	70	2749	194
***Rattus norvegicus***	231	-	1514	39

Summary of the number of validated interactions for each species we have studied as well as the source where this interaction was reported.

**Computationally predicted interactions**. Today, the use of computational methods has sped up considerably miRNA target analysis. Currently available computational methods can be grouped into *ab initio*, machine learning and hybrid methods [15].

*Ab initio *algorithms are based on the experimentally defined rules of miRNA targeting. Among them, MiRanda[23] uses an estimated complementarity score to select the duplexes, MicroTar[24] considers different sequence complementarities in the seed (nt 1-7 and nt 2-8) allowing for G:U wobbles. PITA[13] selects seed matches of six to eight nucleotides, allowing up to one G:U wobble in 7 and 8-mers and up to one mismatch in 8-mers. TargetScan[11] first searches perfect complementarities to the seed and then calculates a score, based on the site type, local A-U enrichment and other aspects of the seed match context. Finally, FindTar[25] finds seed matches allowing up to one G:U wobble and scores them by the position of the central loop. Except TargetScan, these methods consider the thermodynamic stability of duplexes using Vienna RNA package [26]. For instance, RNAhybrid[27] and miRiam[28], first maximize the thermodynamic stability of the miRNA-mRNA pair and then search for sequence complementarities.

Machine learning algorithms, such as RFMirTarget[29] and MultiMiTar[30], filter predictions from *ab initio *algorithms by using classifiers trained with feature patterns extracted from experimentally-validated interactions. RFMirtarget is based on a random forest classifier that evaluates 17 features extracted from a previous prediction performed using miRanda on the test dataset. MultiMiTar is a support vector machine-based algorithm that rewards 90 features of the miRNA-mRNA pair. These features are selected by means of the novel multiobject metaheuristic technique AMOSA[31] integrated with SVM. Both methods were trained 289 interactions extracted from miRecords database and 289 systematically identified tissue-specific negative examples and evaluated using an independent experimentally validated set of interactions.

One example of hybrid methods is NBmiRTar algorithm [32]. It first applies the miRanda algorithm, and then uses a Näive Bayes classifier to filter the output based on 57 features. NBmiRTar was trained with a set of 225 positive miRNA targets of 5 animal species and 38 negative interactions from TarBase.

**Databases of predicted interactions**. Some computationally predicted interactions have been incorporated to different databases: EIMMo[33], DIANA-microT[34], Microcosm [35], http://Microrna.org[36], TargetScan[37], MirDB[38], PITA, miRWalk-predictive [17] and TargetSpy.

Among them, MiRWalk algorithm first searches for perfect complementarities in the seed and then extends it until a mismatch is found. EIMMo searches possible target sites of a microRNA in four different species, retrieves the number of species in which the site is conserved and using Bayes statistics it calculates the probability of conservation of the seed. DIANA-microT searches for 7-, 8- or 9-nt long seed-matches, or 6-nt seeds with one G:U wobble and retrieves the weighted sum of conserved and non-conserved sites of a gene in up to 27 species. Finally, MirDB uses the SVM-based algorithm MirTarget2 [39]. A comparative description of *in silico *prediction methods is shown in Table 2.

**Table 2:** *Comparison of sequence-based algorithms for miRNA-mRNA target predictionn*.

Method^a^	Name	Seed	ΔG	**Conserv**.	Wobbles	ΔΔG	Other features	Type of classifier	Scoring	DB?	software?	website
AI	miRanda	✓	✓	✓			matches with the first 11 nt's of the miRNA are rewarded		score	✓	✓	http://www.microrna.org

AI	TargetScan	✓		✓			different seed types and AU content		score	✓	✓	http://www.targetscan.org

AI	PicTar	✓	✓	✓					score	✓		http://pictar.mdc-berlin.de/

AI	RNA22	✓	✓				miRNA paired to statistically significant patterns in the mRNA					http://omictools.com/rna22-s5063.html

AI	RNAhybrid	✓	✓				MFEs modeled as extreme-value distributed		MDE (energy)		✓	http://bibiserv.techfak.uni-bielefeld.de/rnahybrid/

AI	PITA	✓	✓		✓	✓	1) G:U allowed in 7mer seed		score	✓	✓^b^	http://genie.weizmann.ac.il/pubs/mir07/
							2) G:U, 1 mismatch allowed in 8mer					

AI	EiMMo	✓		✓			model the evolution of orthologous target sites in related species		score	✓		http://www.mirz.unibas.ch/EIMMo3/

AI	DIANA-microT	✓	✓	✓	✓				score	✓		http://diana.cslab.ece.ntua.gr/microT/

AI	MicroTar	✓	✓						p-value		✓	http://tiger.dbs.nus.edu.sg/microtar

AI	FindTar	✓	✓		✓		central loop score to reduce false positives		score and energy		✓	http://bio.sz.tsinghua.edu.cn/

AI	miRiam	✓	✓			✓				✓	✓	http://ferrolab.dmi.unict.it/miriam.html

AI	microcosm	✓	✓	✓			Uses miRanda. Requires: complete seed complementarity and conservation at the same position and in ≥2 species		score	✓		http://www.ebi.ac.uk/enright-srv/microcosm/htdocs/targets/v5/

AI	miRWalk	✓	✓				also a DDBB with experimentally-validated targets from text mining		p-value			http://mirwalk.uni-hd.de/

ML	miTarget	✓	✓				Starting set: miRanda. Radial basis function	SVM				http://cbit.snu.ac.kr/~miTarget/introduction.html

ML	MirTarget2	✓	✓	✓			Initial set: TargetScan, PicTar, miRanda, MirTarget	SVM	score (from probabilities)	✓		http://mirdb.org

ML	TargetSpy	✓	✓	✓			Starting set: PicTar. Generates candidate zones of binding and a representative hybrid (1st or 2nd nt of the miRNA is paired)		score	✓	✓	http://www.targetspy.org

ML	mirSVR	✓	✓	✓			Starting set: miRanda	SVR	mirSVR score (probability for down-regulation)	✓	✓	http://microrna.org

H	NBmiRTar	✓	✓	✓			NB classifier is applied to the output of the miRanda program	Naïve Bayes	NB score (probability)			

a	AI = Ab Initio, ML = Machine Learning, H = Hybrid											

b	Academic use only											

Comparison of different algorithms of miRNA-mRNA target prediction including different algorithm features, the databases and software availability, scoring method, type of classifier, and species for which the algorithm was designed.

### Combination of miRNA-mRNA interactions

One drawback of sequence-based methods is the large numbers of false positives they predict. Some studies have made use of conservation analysis for interaction filtering. However, this can lead to the loss of species-specific interactions.

In the last years, several unions and intersections of different databases have been proposed to improve the specificity and sensitivity of the predictions. One of these attempts was done in [40]. Here the performance of TargetScan, DIANA-microT, miRanda, TargetScanS and PicTar[41], as well as their combinations was compared. The highest value of specificity was obtained for the intersection of the five algorithms and the specificity for the different proposed combinations was over 66.7%.

In [42], authors experimentally analyzed the intersection of the possible microRNA regulators predicted by TargetScan, miRanda and PicTar for the human angiotensin II type 1 receptor (hAT1R). They validated one of the initially considered interactions showing that using the intersection of databases was a viable way of interaction filtering.

Other approaches, such as, ComiR[43], ExprTarget[44], Ranking Aggregation[45], BcmicrO[46], GenMiR3 [47] and a Bayesian Graphical model[48], combine the scores of different databases (see Table S1 in additional file 1).

The aim of GenMiR3 and the Bayesian Graphical model is not to combine different databases but to extract the most outstanding interactions given the miRNA and mRNA expression data as well as sequence based information. However both perform database combination internally and are based on logistic priors.

Ranking Aggregation method is designed to combine different numbers of top-K ranked lists and is based on Cross Entropy Monte Carlo method. It was successfully evaluated in combining the ranked list of targets of human miR-155-5p predicted by miRanda, TargetScan and PicTar.

ComiR combines four databases by estimating the probability of every gene of being targeted by the input set of miRNAs by using an SVM algorithm. If available, miRNA expression data is also considered.

ExprTarget uses a logistic regression to combine the scores of different databases with expression data of mRNAs and miRNAs. In their model, each of the scores of the databases is weighted by means of their capability of replicating experimentally validated interactions. Expression values are used to fit a linear model for each pair and the obtained p-value of the fit is used as an additional score in the model. ExprTarget is based on miRanda, TargetScan and PicTar scores. TarBase is used as gold standard. ROC curves [49] showed that ExprTarget outperforms individual databases.

Finally, BCmicrO uses a probabilistic model to determine how likely is an interaction to be experimentally validated given the scores in different databases. This model is expressed in terms of individual conditional probabilities, one per database and interaction. The authors generated a negative set of miRNA-targets to use as true negatives. This method was tested with TargetScan, miRanda, Pictar, mirTarget, PITA and DIANA-microT. ROC curves showed BCmicrO method outperformed individual databases.

**Our approaches**. Currently, there is no method considerably better than others in predicting microRNA targets. Some recently developed tools provide different ways to combine predictions of several algorithms, assuming that they perform similarly and share the same scoring system, which is not necessarily true. Ideally, an integration of different prediction algorithms should take into account their level of performance as well as the score of each interaction when it is reported by more than one method. In this study, we introduce two complementary approaches to improve the miRNA-mRNA interactions by combining nine predictive algorithms, as well as experimentally validated interactions. Global performance of the algorithms as well as the individual score of every interaction reported by different methods is taken into account. We show that the combination outperforms previous approaches while reducing the number of potential targets candidates.

## Results and discussion

**Reliability of databases**. It is difficult to compare across the different databases of interactions since they differ in size, quality of the interactions and the ability of the scores to reflect the quality of the interactions. We have used a hypergeometric test to measure the reliability of databases (see section "*Measuring the reliability of databases*" in the Materials and Methods section). Results are shown in Table 3.

**Table 3:** *Reliability of databases*.

	(1)	(2)	(3)	(4)	(5)	(6)
**Method**	**Z_score_**	**# int. Z_score_**	**# DDBB**	**# EV**	**# EV / # DB**	**% drawn**

LRS	-89.27	163829	4669137	4286	9.18E-04	9.2

WSP	-84.52	123589	4669137	4286	9.18E-04	6.94

EiMMo	-61.87	191582	1781671	2949	1.66E-03	10.75

DIANA-microT	-54.51	269525	2889574	3010	1.31E-03	11.77

http://www.microrna.org	-21.2	134227	737379	2685	3.64E-03	18.2

microcosm	-17.99	6035	352016	784	2.23E-03	1.71

PITA	-15.2	75683	206722	1425	6.89E-03	36.61

TargetSpy	-14	178114	300000	653	2.18E-03	59.37

miRWalk	-9.92	422089	780000	1243	1.59E-03	54.11

TargetScan	-9.29	19491	132809	1832	1.38E-02	14.68

mirTarget	-5.08	149088	691265	234	3.39E-04	21.57

(1) Minimum z-score of a hypergeometric distribution. The lower the z-score the more statistically significant the enrichment in experimentally validated interactions is. (2) Number of interactions for the minimum z-score. (3) Total number of interactions in the database. (4) Number of experimentally validated (EV) interactions in the database. (5) Proportion of EV interactions within the database. (6) Proportion of selected interactions in the database for the minimum z-score.

This table shows the different databases sorted by z-score that, in turn, is a measure of their enrichment in experimentally-validated interactions when sorted by their provided score. The first two rows of this table correspond to WSP and LRS databases in this work and we will refer to them later. EiMMo and DIANA-microT are top-ranked according to the z-score. TargetScan, although being lowly ranked by the z-score, has the highest proportion of experimentally validated interactions. It seems that TargetScan focuses on including only the most reliable interactions. On the other hand, mirTarget is a medium-sized database, but the quality of interactions, in terms of proportion of experimentally validated interactions, is small, and therefore, lowly ranked in this table. TargetScan results indicate that using z-score as the unique parameter for database comparison is not sufficient (other factors should also be considered, for example, column (6) of table 3). However, this measurement seems reasonable to compare databases with large non-uncut lists of interactions. This is the case of many of the databases used in this work.

**Comparison of methods**. Materials and methods describe in detail the two approaches we propose to combine predictive miRNA-mRNA interactions from nine different algorithms widely used by the scientific community. The first one, named Weighted Scoring by Precision (WSP) is based on summing up the weighted scores for different databases whilst the second one applies logistic regression to find the combined score (LRS). These approaches were evaluated using four different experimentally validated interactions databases to define the tradeoff between sensitivity and specificity. The evaluation of our methods has been restricted to the comparison against the predicted databases and algorithms and compared with two widely used integration methods: the union and the intersection.

**Figure 1:**
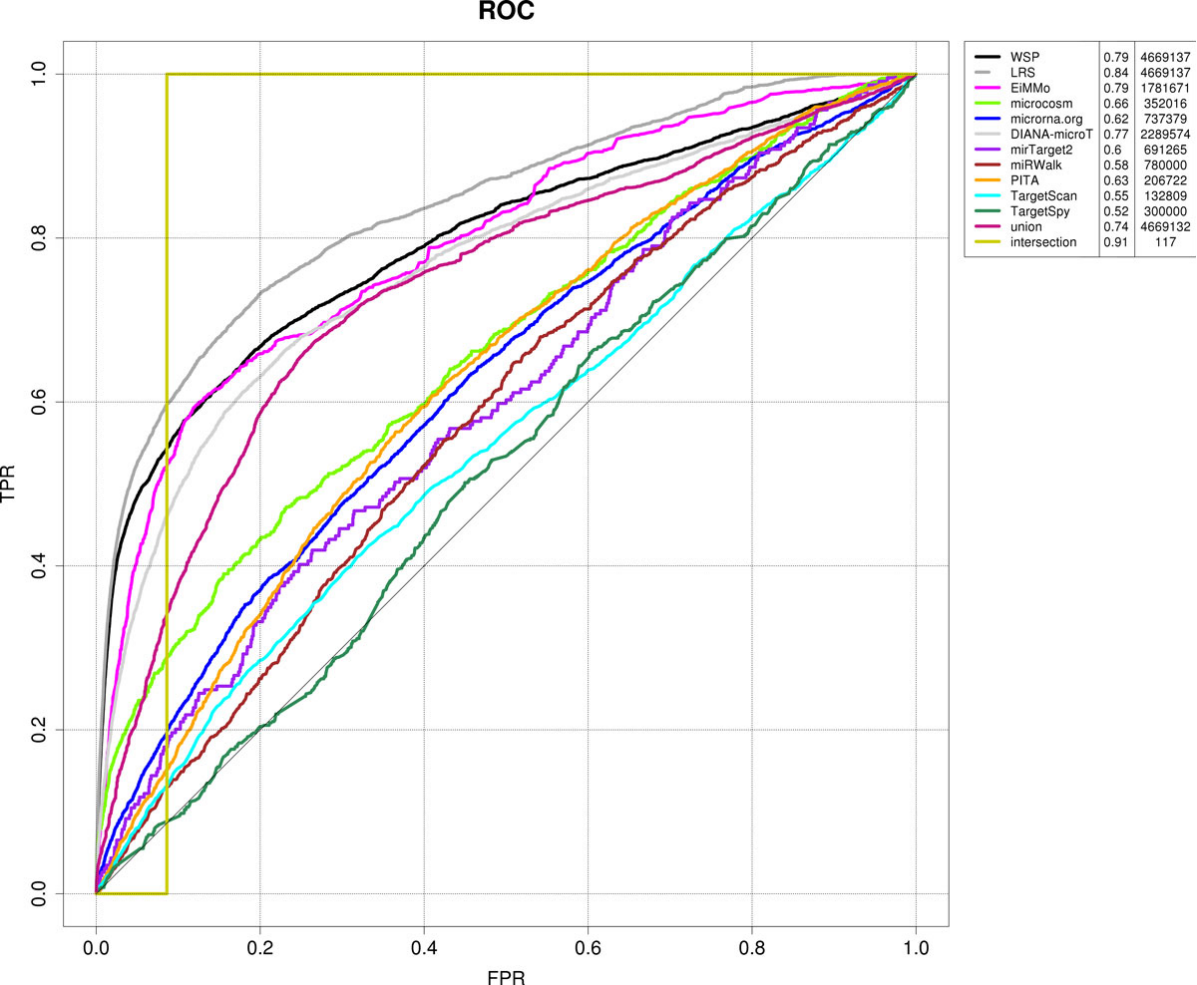
***ROC curve for all predictive algorithms as well as the two combined approaches***. The area under the curve and the number of interactions are also included for every algorithm.

Both approaches outperform any of the predicting algorithms. A first evaluation of the predictions of our two methods has been done using the hypergeometric test used previously for database reliability measurement (Table 3). It can be noted that both of them rank better than any other considered database in terms of z-score and number of interactions. Figure 1 shows the ROC curve of the individual predicting algorithms as well as the two combined approaches we introduced here. LRS outperforms the rest of the algorithms in terms of the ROC curve, while WSP also improves almost all the other algorithms and behaves similarly to EIMMO. It is important to notice that the number of interactions predicted by the different algorithms is quite different, except for the combined approaches that use all miRNA-mRNA pairs reported by the different methods (see Table 3).

**Figure 2:**
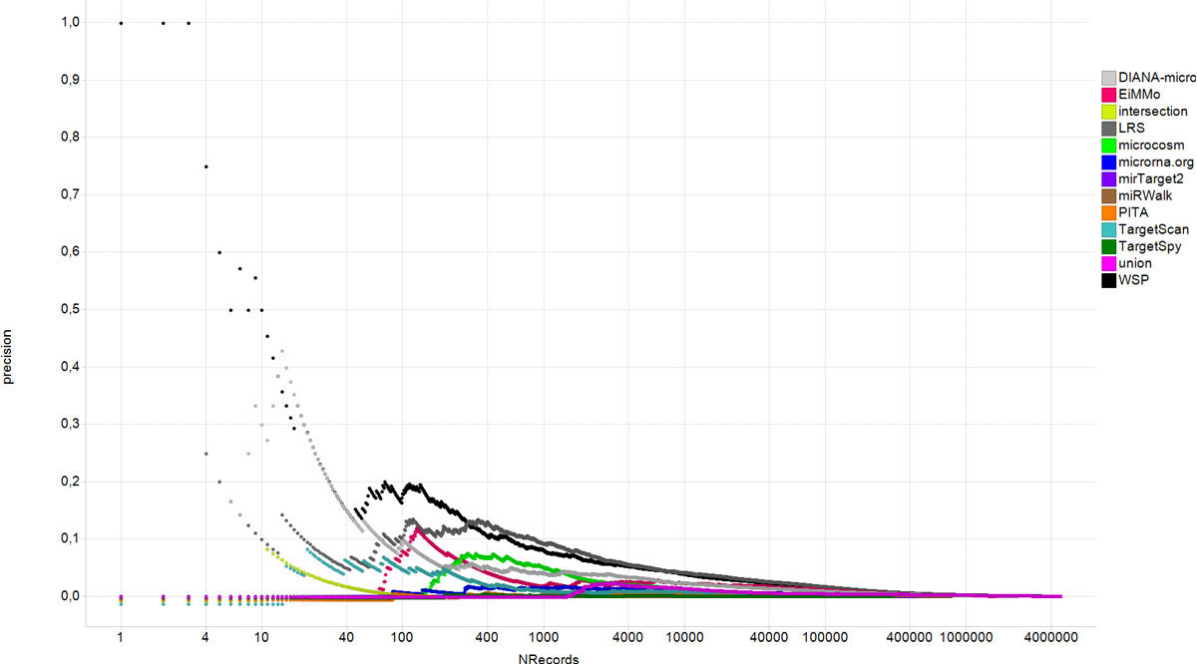
***Precision curve for all predicting algorithms and the combination methods***. The curve was corrected by subtracting the precision value that corresponds to random interactions. The Y axis shows the Precision and the × axis the interactions sorted by score in descending order.

For this particular application, both, the number of False Positives and True Negatives, cannot be exactly estimated. To limit the effect of this missing information, we proposed the use of the precision curve described in Figure 2. The ranking of the different methods resemble those reported in the ROC curve, however, the improvement in performance of the combined approaches is now clearer. Figure 1 shows an example of this effect is ROC AUC of the intersection (0.91), it is much higher than those of the proposed methods (i.e., 0.79 for WSP and 0.84 for LRS). It appears that the intersection is the best-performing method in terms of ROC AUC. This appreciation is misleading. Intersection seems to be the best method since it is the most conservative one. Intersecting all the prediction databases results in only 117 interactions and most of them are obviously experimentally validated. The ROC curve is also misleading because since there are no databases containing non-interactive miRNA-mRNA pairs, the number of False Positives and True Negatives cannot be exactly estimated. There is no optimal solution available for this and that is why we proposed the use of the precision curve described in Figure 2. As can be seen in Figure 2, the intersection is not the most prominent. On the other hand, it is known that if the positive and negative populations are not evenly balanced, the ROC curve does not reflect adequately the behavior of the classifier[49].

Differences between the two proposed approaches are apparent in both the ROC curve and the Precision curves. We noticed, however, that the main inconsistencies concern the first 400 interactions, which represents less than 0.01% of the entire universe of predicted miRNA-mRNA pairs. This is a very small percentage of interactions. Our observation is that the exact score values in the method are not as important as being in the top of the list with a high prediction value. From that perspective, both methods are perfectly compatible. These differences are highlighted in Figure S2 in the supplementary material.

In this work we have compared our methods with the two most used integration and straightforward approaches: the union and the intersection. Although a full comparison with all available methods would be ideal, this is not always possible for several reasons: a) The idea in this contribution is to use the largest amount of individual prediction methods and databases available and therefore the integration needs to be performed with the same databases and algorithms to make a fair comparison. Most of the integration approaches that we cite in the paper use only a subset of the databases and this would make the comparison very unfair. b) Availability of the code or data: most of these methods do not provide a full code we can run and modify or the full interactions data; therefore, a full comparison is in some cases virtually impossible. c) Lack of simple ways to reproduce and calculate these results several times.

Despite of our efforts in making a full comparison, only ExprTarget could be included, even if the comparison was not totally equal in the conditions we have used for the union and intersection. Supplementary section 4 contains all details.

We consider that, as it is already happening in other areas, there is a need of community efforts to provide the data, and algorithms available to facilitate comparison.

All the interactions from the computational methods described in this work, as well as all the experimentally validated interactions are available to the scientific community in a database accessible via web at http://m3rna.cnb.csic.es. Predictions are sorted according the criteria presented here. Each individual prediction is reported with a new combined score. This functionality allows researchers to access a unified repository with most of the available and known information about miRNA-mRNA interactions and use it to compare it with their own methods.

## Conclusions

No miRNA-mRNA algorithm makes perfect predictions under every condition. Because of the multi-faceted nature of miRNA targeting, and the lack of consensus among existing predictions, it makes sense to combine them in a way that maximizes the number of true predicted results while minimizing that of false ones. There have been previous attempts to combine the predictions of several algorithms by first taking their union or intersection, as a way to improve accuracy or coverage, balancing out their sensitivity and specificity, and finally, choosing the most likely candidates by consensus. Most of these approaches give the freedom to choose which combination of prediction algorithms to use. The main issue is that a significant proportion of users do not have the necessary information about each algorithm's performance to make an educated decision.

Our approaches present alternative solutions to this problem by assigning confidence scores to each prediction regardless the algorithm that originally predicts it. Both methods provide a score that objectively quantifies the quality of a particular interaction given its score and the database (or databases) that predict it. This solves the implicit problem of choosing a candidate by consensus in which the confidence of the predictions is not taken into account. In addition, it solves the problem of setting the thresholds (different for each of the prediction databases) to decide whether a predicted interaction is sufficiently reliable or not.

There are some limitations in our approaches that represent open research problems for the scientific community and could be interesting future research directions. For example, we assume that prediction algorithms have a high precision when they contain many validated interactions in their top scores, but this does not necessarily mean that algorithms with low precision are not predicting true interactions. It may just mean that the interactions they predict are harder to prove experimentally, or because the necessary experiments to validate them were never carried out. Almost all prediction algorithms, however, make this assumption. Another weak point in our approaches is that they start with the predictions that were reported by their authors and that are publicly available. It turns out that different reported interaction databases use different versions of sequence databases and therefore, the universe of mRNA used for predictions by the different algorithms is not exactly the same. Rerunning all prediction algorithms with the same mRNA and miRNA sequences would solve this limitation. It is difficult, however, to reproduce the same results than the ones reported on the authors' web sites because of parameters selection and availability of the code. This issue has no simple solution and no statistical tests or algorithms would solve it. We consider that a community effort by the algorithms' providers is the only way to solve this problem, either by making all codes available or by providing updated results of their prediction based on a common set of miRNAs and mRNAs. Our methods presented here do the best they can with the available information and helps in minimizing the negative effect of this lack of homogeneity of the databases. As a final limitation, the lack of information at the transcript level from both predicting algorithms and experimentally validated databases create an important limitation to any method that combines predicting algorithms. Interactions make more sense when they are described at the transcript level, if possible.

As our understanding of miRNA targeting improves and experimental methods become cheaper and more precise, our combined database will become more sensitive and specific. A good example is the starBase database[21]that contains interactions identified by the latest and more precise high-throughput techniques. It will certainly become one of the reference databases for experimentally validated miRNA-mRNA interactions. Integrating starBase, as well as any other new database, will be a future logical continuation of this work and a good input for future versions of M^3^RNA. Besides the databases of experimentally validated miRNA-target interactions, there are quite a few large-scale expression-based analyses which can also be used as alternatives for prediction validation[50]. We have developed these approaches to serve as a useful way to obtain higher-confidence predictions using all available information and thus we hope that new opportunities will span from this.

## Methods

**Heterogeneity of formats and normalization of scores**. Prior to the combination of databases, the heterogeneity of information as well as storing formats must be taken into account. One of such heterogeneities concerns transcript-wise or gene-wise identification of interactions. In order to unify those from different databases, we converted transcript-wise predictions to the gene level. Similarly, we unified gene and microRNA names by translating from one nomenclature to another by using dictionaries. We have used a dictionary between microRNA names from mirbase (ftp://mirbase.org/pub/mirbase/CURRENT/aliases.txt.gz), and translations between different gene names retrieved from Ensembl Biomart. Finally, with the aim of unifying the different scores used in each database, we normalized each score by scaling them to range between 0 and 1, being 1 the highest confidence for the interaction. Another normalization strategy was tested to eliminate the effect of high dense scoring ranges in the original databases. To that end, the scores from each database were substituted by one minus their cumulative density function (cdf) evaluated at the location of the score. In other words, the scores from each database were sorted in an increasing manner and its new score was calculated as one minus their rank, and then divided by the number of interactions. The results with this normalization method do not show any improvement on the approaches we propose here, which reflects their robustness.

**Measuring the reliability of databases**. As a measure of the reliability of the databases, a similar measuring approach to that described in [51] was used. In brief, the reliability is measured as the enrichment in experimentally validated interactions and it is determined from the hypergeometric distribution. Let's assume that E is a set that includes all the interactions predicted by any of the databases with any score. Few interactions in E have been experimentally validated. Each of the databases provides a set of scored interactions. For each database, we sort the interactions according to their scores and we run one hypergeometric test for each threshold in the scores. Finally, we determine the highest enriched set of interactions by selecting the threshold associated to the lowest p-value. The p-value is an indicative of the enrichment in experimentally validated interactions. The lower the p-value the more enriched the selected set will be. Since the p-values are very small, it is likely to have round-off errors and therefore the approximation suggested in [52] was used. Results are included in Table 3.

**Combination of experimental databases**. The experimentally validated interactions have been used here as a gold standard to measure the reliability of predicted interactions. In this work, the union of all the experimentally validated interactions has been considered. A brief description of the databases used can be found in Table 1.

**Evaluating the performance**. The comparison of the performance of our both approaches and the predictive databases was carried out by using the ROC and the Precision curves. The set of the parameters of the ROC curve, number of true positives (TP), number of false positives (FP), number of true negatives (TN) and number of false negatives (FN) have been determined by considering the experimentally validated interactions. In fact, an interaction will be considered to be a: TP in case it has been predicted and validated, a TN in case it has neither been predicted nor validated and a FP or FN in case it has either been predicted and not validated or it has been validated but not predicted.

In machine learning, the area under ROC Curve (AUC) is one of the most widely used approaches for performance measurements. In the ROC curve, the TPR (True Positive Rate) is plot against the FPR (False Positive Rate). The sensitivity or True Positive Rate (TPR) is calculated as: TPR = TP/(TP+FN) while 1-specificity of False Positive Rate (FPR) is calculated as FPR = FP/(FP+TN). Each point in a ROC curve is obtained by setting different threshold values to the normalized scores. This threshold is varied in decreasing order, from 1 (most stringent) down to 0 (more relaxed). Figure 1 shows the ROC curve for all predictive algorithms used in this study as well as the two integrating approaches described here.

This evaluation approach has some caveats. First, not all real interactions have been experimentally validated. And secondly, in general, databases with experimentally validated interactions do not include tested and not validated interactions. Therefore, some of the false positives and almost all true negatives will be erroneously labeled. This implies that the ROC curve can produce to unavoidable misleading evaluations.

An alternative approach could be to use the Precision-Recall curve (PR). In this curve, the Precision = TP/(TP+FP) is plotted against the TPR, also known as recall. However, it has been shown that an approach that dominates in the ROC space it also dominates in the PR space [53]. Hence, the Precision-Recall curve also suffers from the same restrictions.

To cope with this situation and to complement the information from the ROC curve, we introduce the Precision curve. For every database, the normalized scores are sorted in descending order and the accumulated precision values are determined. The resulting curve shows the fraction of the predicted interactions that have been experimentally validated versus the number of predicted interactions. This approach is not immune to the potential missing information since there is still a dependency of false positive values, which cannot always be estimated. However, the effect of true negatives is not taken into account, which minimizes an important source of missing information. Figure 2 shows the precision curve for all predicting algorithms as well as for two combination methods described in this manuscript.

Concerning the evaluation of the LRS method using the ROC curve it is important to point out that the model could be overestimated, since the same experimentally validated interactions have been used for both the prediction and the evaluation. However, given that the number of parameters used in the model is much smaller than the number of interactions, this overestimation is not expected to be large. In fact, using glmnet [54] the R package that internally performs cross-validation to find out the values of the regressors, we got a very similar AUC (0.84 using all the data vs. 0.836 using cross-validation) (see supplementary section 3).

**Figure 3:**
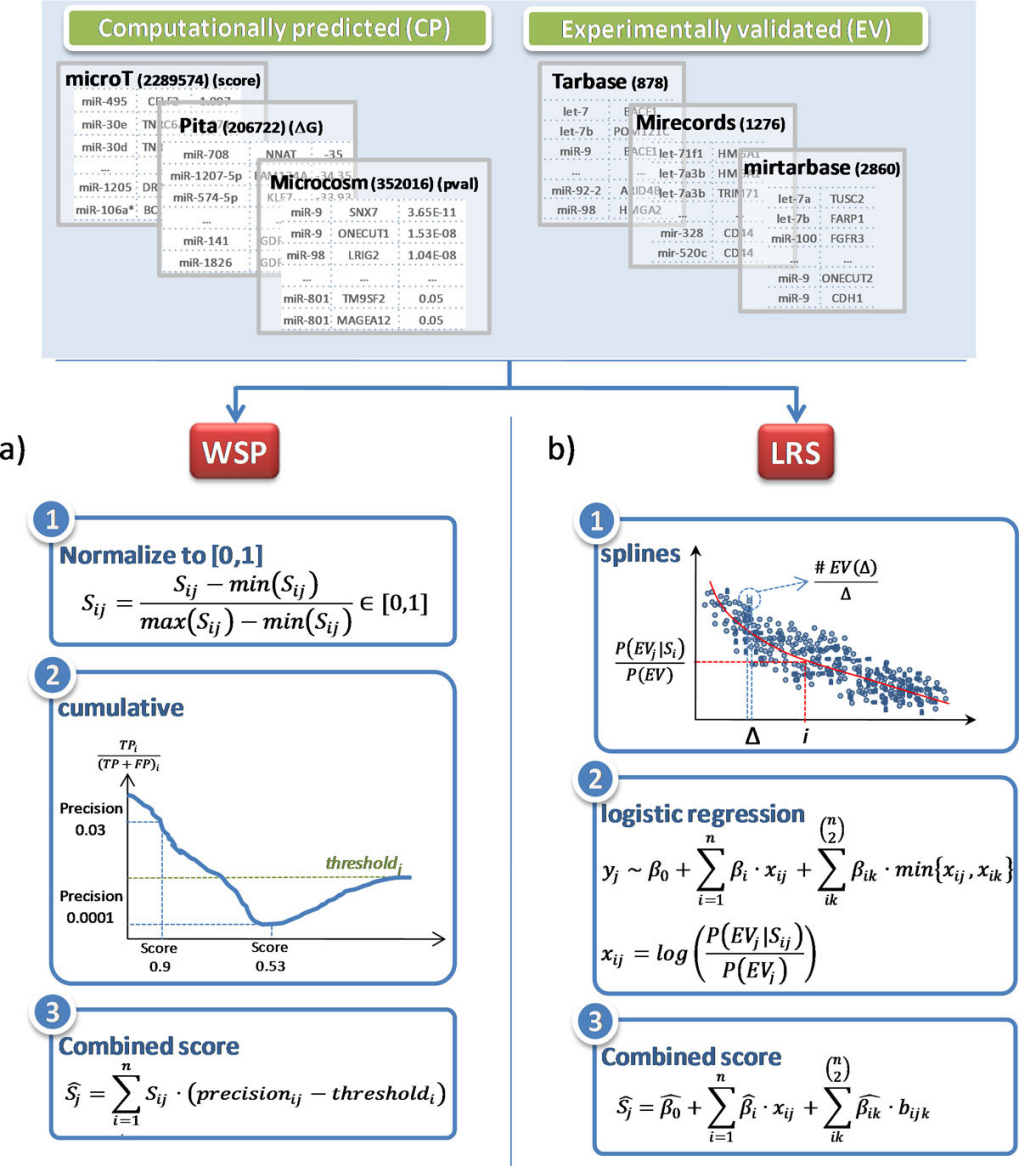
***Description of the WSP and LRS methods***. In the WSP method, box a), a new score for each interaction in each database is calculated by weighting their original scores with their associated accumulated precision. To this aim, for each of the databases, the interactions are sorted and their corresponding accumulated precisions are calculated. The obtained precision values are considered to be reliable in case they are larger than the randomly expected precision of the database. In the LRS method, box b), each interaction in each database is re-scored by assigning its probability of being experimentally-validated. To this aim, for each database, the probability of each interaction of being experimentally-validated is calculated. The probabilities in different databases are then combined considering their possible dependencies.

**Approach #1. Weighted scoring by precision (WSP)**. The WSP method combines the scores of each interaction in different databases by calculating a weighted sum of their normalized individual scores (see Figure 3.a)). The weights are included to consider that the scoring methods used in different databases are not equally reliable. To this aim, the interactions in each database are sorted from the best to the worst and then, the accumulated precision (TP/(TP+FP)) for each of the positions in the sorted list (each interaction) is determined. The accumulated precision for one interaction takes into account the number of TPs and FPs from the first interaction. In order to account for the reliability of the database, the precision value of each interaction is corrected by subtracting the expected precision value for the database, obtaining positive values only for those interactions that are not performing similarly to randomly selected ones. Each new integrated score is then calculated as the sum of each individual score from each database multiplied by the precision of that interaction in the specific database (weight).

With this method, highly re-scored interactions will be those that: a) have been highly scored in individual databases, b) are more likely to be experimentally validated and c) have been predicted with high score in many of the predictive databases. This global score is robust to the incorporation of low-performing scores, i.e. databases with low-performing score will not drag down good scoring ones.

**Approach #2. Logistic Regression combined Scoring (LRS)**. The LRS method assumes that the higher the probability of an interaction of being experimentally validated, the higher its reliability is. This method, first, determines the probability of each interaction in each database of being experimentally validated and then, combines them to get for each interaction a single probability by using a logistic regression model. The steps, detailed in Figure 3.b) are the following: 1) interactions in each database are ranked according to their scores, then 2) the scored list of each database is divided into a number of bins for which the fraction of the number of experimentally-validated interactions is determined, 3) the obtained set of points is interpolated using smoothing splines and 4) finally, these new scores are combined using a logistic regression model.

Figure S1 of the supplementary materials shows the interpolating splines for each of the databases that in turn, provide the probability of each interaction in a database of being experimentally validated. The proposed logistic regression model is equivalent under some conditions to a probabilistic model (see supplementary material). The logistic model includes cross-terms across the databases to accommodate possible redundancies in their information.

**miRNA-mRNA database: m^3^RNA**. The combined databases obtained with both methods have been included into a web page (also accessible via webservices). This webservice also includes the computationally predictions interactions from the different databases used here. miRBase and Ensembl names have been used as the reference name for miRNAs and genes and transcripts respectively.

The database has been implemented using postgreSQL, and all operations and accessions are managed by a Ruby interface. This interface is connected to a SOAP webservices server to provide a remote programmatic access allowing read only operations. Users can access the information within the webservice by providing the organism and a list of miRNAs and/or genes. Data is returned in table format with: the combined databases, names of the miRNAs and mRNAs involved in each interaction, experimental information in case available and, the normalized scores and precisions for every predictive algorithm.

To access the information in a more friendly manner, we have created a website on top of the webservices using the web application framework Ruby on Rails. Further information can be found in the "Help" section of the web page. The database is available at http://m3rna.cnb.csic.es

## Availability of supporting data

M^3^RNA website is freely available on the web at http://m3rna.cnb.csic.es/

## Competing interests

The authors declare that they have no competing interests.

## Authors' contributions

DTM and ISC developed the code and simulations for the WSP method. AM developed the code and simulations for LRS method. DJMH collected information and data from all available predicting and experimental interaction databases. COSS provided insights in the statistical evaluations. DTM, ISC, DJMH and AM developed and implemented all methodologies described in this study. DTM developed the m^3^RNA. APM and AR conceived the idea, designed experiments and supervised the project. All of the authors participated in the redaction, read and approved the final manuscript.

## Supplementary Material

Additional File 1 **Contains a table with a brief description of methods for the combination of miRNA-mRNA interactions from different databases, figures of the alternative and discarded scoring normalization method for predictive databases, a mathematical description of the LRS method, cross validation results to test overfitting of the model and comparison with other integration methods**. Click here for file

## Declarations

The authors acknowledge support of the publication fee by the CSIC Open Access Publication Support Initiative through its Unit of Information Resources for Research (URICI).

This article has been published as part of *BMC Genomics *Volume 15 Supplement 10, 2014: Proceedings of the 25th International Conference on Genome Informatics (GIW/ISCB-Asia): Genomics. The full contents of the supplement are available online at http://www.biomedcentral.com/bmcgenomics/supplements/15/S10.
