# Supplementary material for: Improving miRNA-mRNA interaction predictions
Source: BMC Genomics. 2014 Dec 12;15(Suppl 10):S2. doi: 10.1186/1471-2164-15-S10-S2 (PMC4304206; doi:10.1186/1471-2164-15-S10-S2)
Supplement: Additional File 1 — Contains a table with a brief description of methods for the combination of miRNA-mRNA interactions from different databases, figures of the alternative and discarded scoring normalization method for predictive databases, a mathematical description of the LRS method, cross validation results to test overfitting of the model and comparison with other integration methods. [file 1471-2164-15-S10-S2-S1.docx]

# Supplementary figures and tables

## Table S1. A brief description of methods for the combination of miRNA-mRNA interactions from different databases.

| **Name** | **Ref.** | **Description** |
| --- | --- | --- |
| Ranking aggregation | [1] | It uses a Cross Entropy Monte Carlo (CEMC) algorithm that iteratively searches the optimal combined list that minimizes a certain criterion |
| Bayesian Network classifier | [2] | The features measured by individual target prediction algorithms are classified and selected to create a new combined list of interactions. |
| ComiR | [3] | It is divided into two steps: 1) re-scoring of miRNA-mRNA interactions and 2) combining them using SVM. Re-scoring is done as follows:   - In case the scores are given as energy values a thermodynamic model based on the Fermi-Dirac equation together with miRNA expression is used   $S_{k}=\sum_{i=1}^{N} \sum_{j=1}^{n_{ik}} \frac{1}{1+e^{(E_{ijk}-\mu)/(RT)}}$  where$E_{ijk}=-RT\cdot ln(K_{i})$ is the energy of the duplex, $\mu=RT\cdot ln([{miR}_{i}])$ and S_k_ is the combined score for gene *k* given microRNA *i*, their binding sites *j* and their concentration values *[miR_i_]*.   - In case interactions are ranked with scores, the new scores are determined by,   $S_{k}=\sum_{i=1}^{N} S_{ik}\cdot[{miR}_{i}]$  where S_k_ is the score associated to miRNA*i* and mRNA *k*. |
| ExprTarget | [4] | A logistic regression model with *x_k,i_* the predictors (the scores of different databases plus the p-values of an adjusted linear regression model between miRNA and mRNA expressions) and the set of experimentally validated interactions as observations,  $log\left( \frac{p_{i}}{1-p_{i}} \right)=\beta_{0}+\beta_{1}\cdot x_{1,i}+\beta_{2}\cdot x_{2,i}+\ldots+\beta_{k}\cdot x_{k,i}$  where*p_i_* is the probability of miRNAi to be real target given the scores *x_k,i_* in databases k. With the obtained *β*-s, the *p_i_* can be determined from,  $p_{i}=\frac{1}{1+e^{-\left( \sum_{k} \beta_{k}\cdot x_{k,i} \right)}}$ |
| GenMiR3 | [5] | Extension of GenMiR++ that adds sequence-based information to estimate *π*, the prior probability of being real target. Given N sequence features represented by N-dimensional vectors $\boldsymbol{f}_{\boldsymbol{gk}}$ and unknown weights $w_{n}$, its prior is set to,  $\pi_{gk}=P\left( s_{gk}=1\vert c_{gk}=1,\boldsymbol{f}_{\boldsymbol{gk}},\boldsymbol{w} \right)=\frac{1}{1+e^{(-\boldsymbol{w}^{\boldsymbol{T}}\cdot\boldsymbol{f}_{\boldsymbol{gk}})}}$ |
| BayesianGraphicalmethod | [6] | Different scores $s_{gm}^{k}$ for interaction $r_{gm}$ are considered in the following prior, where $\boldsymbol{\tau}$ is an unknown variable  $P\left( r_{gm}=1\vert\boldsymbol{\tau} \right)=\frac{1}{1+e^{-(\mu+\tau_{1}\cdot s_{gm}^{1}+\tau_{2}\cdot s_{gm}^{2}+\ldots++\tau_{k}\cdot s_{gm}^{k})}}$ |
| BCmicrO | [7] | The aim is to determine the probability $P\left( y=1\vert x_{1},x_{2},\ldots,x_{k} \right)$ of an interaction of being real *y=1* given the scores *x_k_* in different databases. The posterior probabilityassumedthatconditionals are independents,  $P\left( y=1\vert x_{1},x_{2},\ldots,x_{k} \right)=\frac{\left[ \prod_{k} P\left( x_{k}\vert y=1 \right) \right]\cdot P\left( y=1 \right)}{\left[ \prod_{k} P\left( x_{k}\vert y=0 \right) \right]\cdot P\left( y=0 \right)+\left[ \prod_{k} P\left( x_{k}\vert y=1 \right) \right]\cdot P\left( y=1 \right)}$  The values of the different probabilities in the equation are determined from experimentally validated datasets. |

## Figure S1. Distribution of the proportion of experimentally validated interactions within a set of interactions with similar score. The y-axis are identical for all the graphs. A point with large y-value indicates a set of interactions with similar scores with many experimentally validated interactions. The red line is a smoothing robust spline [8] that interpolates the cloud of points. The value of the spline is expected to be the probability of being experimentally validated given the score in each database.

## Figure S2. Precision curves for the two combined approaches presented in this work. a) Precision curve for WSP based on the weighted sum of interactions. b) Precision curve for LRS. The labels of the top miRNA-mRNA pairs are shown in both cases.

# Description of LRS method

In this section of the supplementary materials, the mathematical formulation of the Logistic Regression Scoring Method is described. Its aim is to predict the probability for a particular interaction of being experimentally validated. This probability is used as a score to rank the interactions. In order to reach this aim the following steps are used:

1) Each database is sorted according to its score (best interactions are first).

2) Interactions are grouped according to the score and for each group; the ratio between the number of experimentally validated interactions in the group versus the group size is determined,

3) These ratios are interpolated using constrained smoothing robust splines[8], and finally

4) A logistic regression is fitted using the scores provided in the splines, taking into account that the same interactions can be given a different score by different databases. The returned log odds of the logistic regression are the new scores that combine all the databases.

In the following paragraphs these steps are further explained.

## Constrained Splines

The first step of the method consists on ranking the scores in each database from the best to the worst score. The ranking of the scores in each database is done by accounting to the type of score: p.values, binding energies or scores. Depending on the nature of the score, the best interactions have the largest or the lowest scores. Ranked list of interactions are then divided into bins and the proportion of validated interactions for each bin is computed. Observe that these proportions can be considered as an estimation of the probability of an interaction in the bin to be experimentally validated. Then, for each of the databases, the obtained probabilities are interpolated using constrained splines. Since the smoothed splines must represent a probability value and are sorted by their scores, the spline is constrained to be 1) bounded by 0 and 1, and 2) be non-increasing. Although other methods such as lowess or loess regression could have been used we decided to use the cobs library due to its versatility, i.e. automatically selects the number of knots and allows adding constrains in both the values and in the derivatives of the spline.

The initial distribution of points (position vs ratio) as well as the spline for each of the databases is plotted in figure S1. These curves reflect somehow the reliability of the scoring method in each database. As indicated in the main manuscript, it has been assumed that for a good database, setting a proper threshold, they have many interactions that are experimentally validated.

## Score combination

The estimated probabilities are a new score that can be compared across the different databases. Since there are interactions, with different scores, provided by different databases, we have taken a probabilistic approach to combine the scores that is further refined by a logistic regression.

Let us assume that *n* is the number of databases with miRNA-mRNA interaction data and let be S_ij_ the score of interaction *j* in database *i*. Then, the probability of an interaction *j* of being experimentally-validated (EV), $P\left( {EV}_{j}|S_{1j}\bigcap S_{2j}\bigcap\ldots\bigcap S_{nj} \right)$, can be mathematically expressed in terms of known probabilities $P\left( {EV}_{j}|S_{ij} \right)$. These probabilities are the ones obtained with the fitted splines in the previous step.By applying the properties of conditional probability and *considering that all databases are independent*,

$$P\left( {EV}_{j}|\bigcap_{i=1}^{n} S_{ij} \right)=P\left( \bigcap_{i=1}^{n} S_{ij}|{EV}_{j} \right)\cdot\frac{P\left( {EV}_{j} \right)}{P\left( \bigcap_{i=1}^{n} S_{ij} \right)}=\left( \prod_{i=1}^{n} P\left( S_{ij}|{EV}_{j} \right) \right)\cdot\frac{P\left( {EV}_{j} \right)}{P\left( \bigcap_{i=1}^{n} S_{ij} \right)}=$$

$=\left( \prod_{i=1}^{n} P\left( {EV}_{j}|S_{ij} \right)\cdot\frac{P\left( S_{ij} \right)}{P\left( {EV}_{j} \right)} \right)\cdot\frac{P\left( {EV}_{j} \right)}{\prod_{i=1}^{n} P\left( S_{ij} \right)}=P\left( {EV}_{j} \right)\cdot\left( \prod_{i=1}^{n} \frac{P\left( {EV}_{j}|S_{ij} \right)}{P\left( {EV}_{j} \right)} \right)$ (1)

In case an interaction is not included in a database, the probability $P\left( {EV}_{j}|S_{ij} \right)$ is set to the probability of an interaction that do not appear in that database of being experimentally validated, i.e. the number of predicted interactions over the total number of interactions not included in the database.

Applying logarithm properties,

$log\left( P\left( {EV}_{j}|\bigcap_{i=1}^{n} S_{ij} \right) \right)=log\left( P\left( {EV}_{j} \right) \right)+\sum_{i=1}^{n} log\left( \frac{P\left( {EV}_{j}|S_{ij} \right)}{P\left( {EV}_{j} \right)} \right)$ (2)

Since the number of experimentally validated interactions is small compared to the large amount of computationally-predicted interactions, the probability $p_{j}=P\left( {EV}_{j}|\bigcap_{i=1}^{n} S_{ij} \right)$is usually small. Thus, the simplification $log\left( p_{j}/\left( 1-p_{j} \right) \right)\sim log\left( p_{j} \right)$ holds. This way the equation above can be viewed as the mathematical representation of a standard logistic regression $y_{j}\leftrightarrow log\left( \frac{p_{j}}{1-p_{j}} \right)=\beta_{0}+\sum_{i=1}^{n} \beta_{i}\cdot x_{ij}$ in where $\beta_{0}$ is equal to $log\left( P\left( {EV}_{j} \right) \right)$, all $\beta_{i}$ are equal to 1 and $x_{ij}$ are equal to $log\left( \frac{P\left( {EV}_{j}|S_{ij} \right)}{P\left( {EV}_{j} \right)} \right)$.

The main advantage of considering this logistic regression is that the independence assumption is no longer needed: the coefficients of the regression will adapt to better represent the data. On the other hand, since all the databases are based mainly on similar approaches (sequence complementarity, binding energy, mRNA secondary structure and so on), they cannot *a priori* be considered independent.

In order to include possible dependencies among the databases, we have extended the design matrix of the logistic regression with additional columns that account for two-way cross-terms of the databases of predictions. In generalized linear models, these new terms are known as *interactions*. However, here they will be termed as cross-terms so as to make the text more understandable, i.e. the term interactions will be restricted here to miRNA-mRNA interactions.

The presence of a cross-term implies lack of independence. Among the possible ways to augment the matrix of scores to include cross-terms we chose the following. We included$\left( \begin{matrix} n \\ 2 \end{matrix} \right)$ new columns (all the two-way cross-terms) with values $b_{ijk}=min\left( x_{ij},x_{kj} \right)$. Using these considerations, the logistic regression for a given interaction is,

$y_{j}⟷log\left( \frac{p_{j}}{1-p_{j}} \right)=\beta_{0}+\sum_{i=1}^{n} \beta_{i}\cdot x_{ij}+\sum_{ik\in\left\{ 1\ldots\left( \begin{matrix} n \\ 2 \end{matrix} \right) \right\}} \beta_{ik}{\cdot b}_{ijk}$. (3)

With this selection if the $b_{ijk}$ coefficient is zero, there is no interaction. If $b_{ijk}$ is -1, the term that corresponds to the “worst” database and the cross-term cancel out and the probability is equal to the largest probability. The expected values for values $b_{ijk}$ are between these 0 and 1 since if the interaction appears in several databases the probability of being experimentally validated is expected to increase. Therefore, with this selection of the design matrix, the expected values of the estimates are:

1. $\beta_{0}$ will tend to $log\left( P\left( EV \right) \right)$,
2. $\beta_{i}$will be close but smaller than 1. The reasoning is the following: in case an interaction is predicted by two databases, its probability of being EV will be higher than the probability in each database but lower than in case both databases are independent,
3. $\beta_{ik}$will be bounded by 0., in case both databases are independent, and -1, in case one of the databases includes the other.

Hence, if two databases are redundant, the expected values of $\beta_{i}$will be smaller than 1 and $\beta_{ik}$will probably be negative. In the extreme case in which the same database is included twice (namely database *i* and database *k*) any solution in which $\beta_{i}+\beta_{k}+\beta_{ik}=1$ would be valid. In order to prevent these cases, we solved the logistic regression using a small regularization term to prevent the inflation of the cross-terms in the logistic regression and stabilizing the coefficients using glmnet package [9].

Finally, the scores of the combined database are determined as follows,

$\hat{S_{j}}=log\left( p_{j} \right)\sim\hat{\beta_{0}}+\sum_{i=1}^{n} \hat{\beta_{i}}\cdot x_{ij}+\sum_{ik\in\left\{ 1\ldots\left( \begin{matrix} n \\ 2 \end{matrix} \right) \right\}} \hat{\beta_{ik}}{\cdot b}_{ijk}$. (4)

# Cross Validation of LRS results

Since in the WSP and LRS methods the same experimentally validated interactions are used for both prediction and evaluation of the combined database, the performance results shown in the ROC could be overestimated. While in the case of WSP this is not critical, since there is no model estimation process, this situation could affect the results of LRS.

In LRS, the number of parameters in the model is very small compared with the number of interactions and thus, the estimated AUC is expected not be too positively biased. Furthermore, LRS model was ran using the R package glmnet (used to estimate the parameters of generalized linear models) that internally performs different cross-validations to find out the values of the regressors and therefore the overestimation effect is intrinsically minimized.

In order to test that the results of LRS method are not overestimated, we did cross validation by using cv.glmnet function of glmnet package[9]. This function retrieves the cross validation results (in our case, the AUC value) for different values of the regularization parameter used in the model. The results are shown in figure S3.


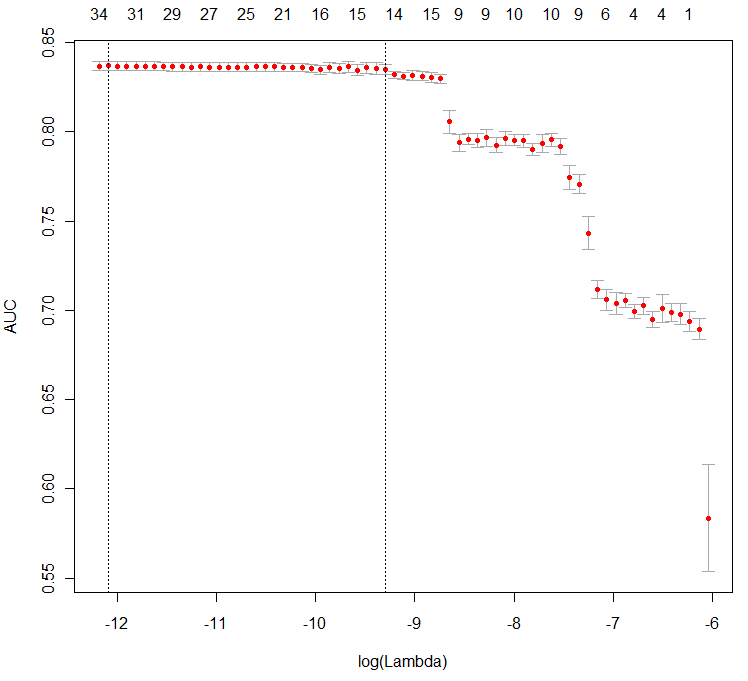


**Figure S3.** Cross Validation results obtained with cv.glmnet of R package glmnet. The figure shows the obtained AUC values for the different values of the regularization parameter used in the cross validation.

The LRS results shown in the paper have been estimated using the lowest regularization parameter. Thus, the AUC shown in the manuscript is comparable to the AUC for the lowest Lambda in the figure S3. Both values are very similar (0.84 vs. 0.836 respectively). This is a proof that the model is not over estimating the experimentally validated database.

# Comparison with other integration methods

In the main manuscript we have used for the comparison the two most used integration and straightforward approaches: the union and the intersection. Although a full comparison with all available methods would be ideal, this is not always possible for several reasons:

- The idea in this contribution is to use the largest amount of individual prediction methods and databases available and therefore the integration needs to be performed with the same databases and algorithms to make a fair comparison. Most of the integration approaches that we cite in the paper use only a subset of the databases and this would make the comparison very unfair.
- Availability of the code or data: most of these methods do not provide a full code we can run and modify or the full interactions data, Therefore, a full comparison is in some cases virtually impossible. In details:
  1. GenMiR3, Bayesian Graphical Method and ComiR are focused in extracting the main interactions that take place in a particular experiment, i.e. their results are tailored to each experiment due to the expression data used. Thus, their predictions are not universal and cannot be applied to other experiments.
  2. The link indicated in the paper of BcmicrO seems to be broken.
  3. There is no downloadable code for ExprTarget. There is, however, a downloadable database of ExprTarget results called ExprTargetDP.
  4. We found that the Ranking Aggregation method is the only one with available code (in the topklists package in R http://topklists.r-forge.r-project.org ). However, when using the full set of interactions, we experienced severe memory issues, which made the analysis impossible.
- Lack of simple ways to reproduce and calculate these results several times.

Despite of our efforts, only ExprTargetDB could be included in the comparison. However, the following must be taken into account. First, the model uses expression data for database combination. As we showed in our previous publication [10], adding expression data to sequence-based prediction enriches the results in experimentally-validated interactions. Second, ExprTarget only uses miRanda, PicTar and TargetScan databases while our approaches use many others. A fair comparison would require all methods to be run under the same conditions: adding or not expression data and including the same set of interactions. In any case, even if the comparison is not totally equal, we evaluated ExprTarget and the results are shown in figures S4 and S5 below.

From the results we can conclude that ExprTarget seems to score very well those interactions that are experimentally validated, however, its performance decreases drastically with the score. The AUC of the ROC curve reflects that it does not perform better than our proposal with the same data. The PC curve, however, shows a drastic improvement over all methods, which it is explained by the dominant effect of the first interactions, most of them experimentally validated.

**Figure S4.** ROC curves for WSP, LRS and ExprTarget as well as for the databases used in the combination.


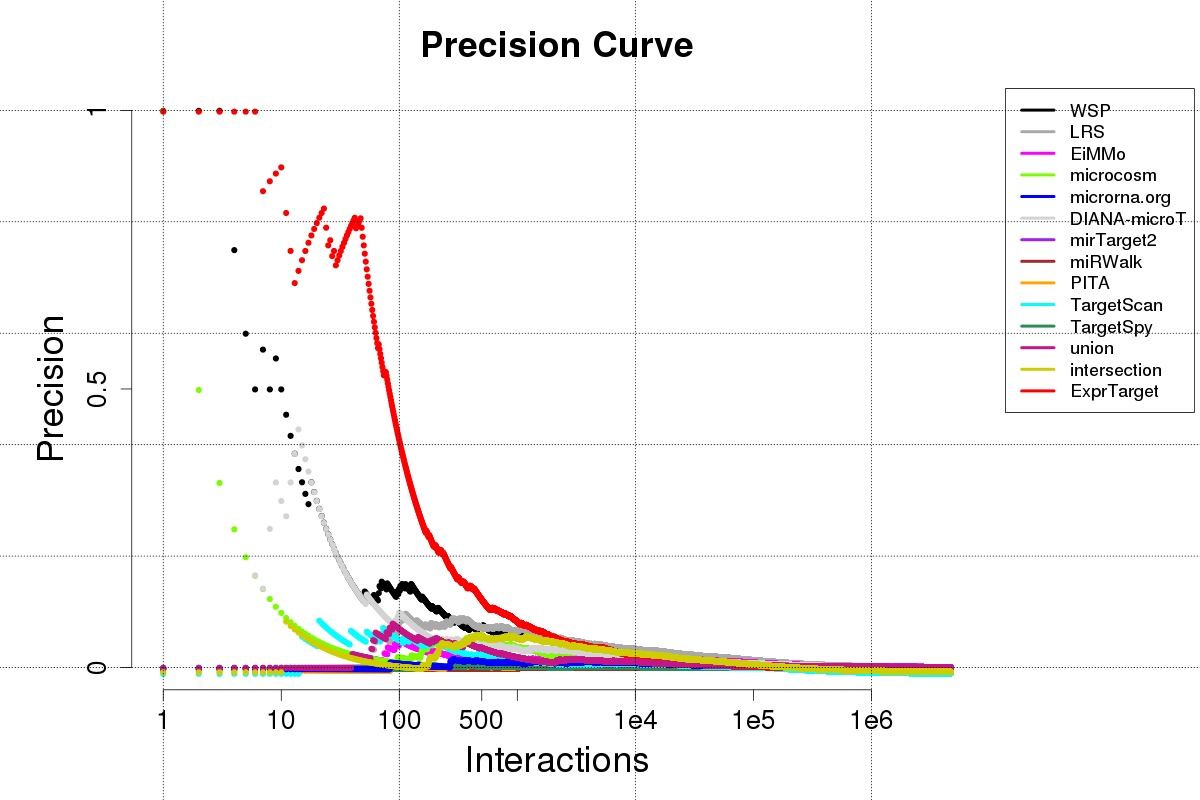


**Figure S5.** Precision curves for WSP, LRS and ExprTarget as well as for the databases used in the combination.

# Supplementary References

1. Lin S, Ding J: **Integration of ranked lists via cross entropy Monte Carlo with applications to mRNA and microRNA Studies.***Biometrics* 2009, **65**:9–18.

2. Zhang Y, Verbeek FJ: **Comparison and integration of target prediction algorithms for microRNA studies.***J Integr Bioinform* 2010, **7**:1–13.

3. Coronnello C, Hartmaier R, Arora A, Huleihel L, Pandit K V, Bais AS, Butterworth M, Kaminski N, Stormo GD, Oesterreich S, Benos P V: **Novel modeling of combinatorial miRNA targeting identifies SNP with potential role in bone density.***PLoS Comput Biol* 2012, **8**:e1002830.

4. Gamazon ER, Im H-K, Duan S, Lussier YA, Cox NJ, Dolan ME, Zhang W: **Exprtarget: an integrative approach to predicting human microRNA targets.***PLoS One* 2010, **5**:e13534.

5. Huang JC, Frey BJ, Morris QD: **Comparing sequence and expression for predicting microRNA targets using GenMiR3.***Pac Symp Biocomput* 2008:52–63.

6. Stingo FC, Chen YA, Vannucci M, Barrier M, Mirkes PE: **A Bayesian graphical modeling approach to microrna regulatory network inference.***Ann Appl Stat* 2010, **4**:2024–2048.

7. Yue D, Guo M, Chen Y, Huang Y: **A Bayesian decision fusion approach for microRNA target prediction.***BMC Genomics* 2012, **13 Suppl 8**:S13.

8. Ng P, Maechler M: **A fast and efficient implementation of qualitatively constrained quantile smoothing splines**. *Stat Modelling* 2007, **7**:315–328.

9. Friedman J, Hastie T, Tibshirani R: **Regularization Paths for Generalized Linear Models via Coordinate Descent.***J Stat Softw* 2010, **33**:1–22.

10. Muniategui A, Nogales-Cadenas R, Vázquez M, L Aranguren X, Agirre X, Luttun A, Prosper F, Pascual-Montano A, Rubio A: **Quantification of miRNA-mRNA interactions.***PLoS One* 2012, **7**:e30766.
